# Use of SGLT‐2 inhibitor in COVID‐19: A cautionary tale

**DOI:** 10.1002/mco2.45

**Published:** 2021-01-28

**Authors:** Zohaib Yousaf, Waqar Munir, Riyadh Ali Mohammed Hammamy

**Affiliations:** ^1^ Department of Medicine Hazm Mebaireek General Hospital Hamad Medical Corporation Qatar

Dear Editor,

SGLT‐2 inhibitors are oral antidiabetic agents with a low risk of hypoglycemia. They block glucose reabsorption at the proximal tubule, leading to glucosuria and thus lowering plasma glucose levels. SGLT‐2 inhibitors have an added benefit of weight loss and a reduction of blood pressure. Diabetic ketoacidosis (DKA) is a serious condition resulting from a relative insulin deficiency in the body and is characterized by marked hyperglycemia and ketoacidosis. Patients on sodium‐glucose co‐transporter‐2 (SGLT‐2) inhibitors have a two‐fold higher risk of developing DKA shortly after initiation, compared to dipeptidyl peptidase‐4 (DPP4) inhibitors.^1^ Furthermore, they may suffer from ketoacidosis in the absence of profound hyperglycemia due to urinary glucose loss.^2^ We stress this point by describing a case of euglycemic DKA in a COVID‐19 patient, where Kussmaul breathing mimicked respiratory distress. This project has been approved by the medical research council of Hamad Medical Corporation under the project ID MRC‐04‐20‐636.

A 49‐year‐old male, known case of type 2 diabetes mellitus diagnosed 5 months back, presented with fever, fatigue, mild dry cough, and flu‐like symptoms. He had exposure to a coworker with COVID‐19. He was a non‐smoker and did not use alcohol or any illicit drugs. He had normal vital signs and an unremarkable clinical examination. A SARS‐CoV‐2 reverse transcriptase‐polymerase chain reaction employing the GeneXpert technique from a combined nasopharyngeal and throat swab was sent and turned out to be positive. His complete blood count, electrolytes, inflammatory markers, liver, and renal function were within normal limits. A random blood sugar of 14.8 mmol/L was noted, and HbA1C was 8.9%. The patient's home medications consisted only of metformin 1 g twice daily. Dapagliflozin 10 mg daily was added to his oral antidiabetic regimen. He was discharged after 24 hours of observation on as needed cough suppressant and acetaminophen.

Five days after his discharge, the patient presented to the hospital again complaining of worsening fatigue, nausea, and shortness of breath. A review of systems, including the cardiac and gastroenterology symptoms, was unremarkable. Vitals showed tachycardia (119 beats per minute), tachypnea (25 breaths per minute), no hypotension or desaturation, and random blood sugar of 6.3mmo/L. Examination showed a middle‐aged dehydrated, anxious‐looking gentleman, sitting up in bed and appearing in mild distress with rapid shallow, labored breathing. The rest of the examination was unremarkable. An ECG showed sinus tachycardia, and a chest X‐ray revealed bilateral perihilar infiltrates. Initial differential diagnosis included COVID‐19 pneumonia‐causing respiratory distress, acute pulmonary embolism, or underlying myo‐pericardial involvement secondary to COVID‐19.

An arterial blood gas revealed a high anion gap metabolic acidosis with a normal lactate and normal renal function (Table 1). He was diagnosed as euglycemic DKA (euDKA) secondary to dapagliflozin and was managed in the critical care unit for 3 days. He received IV fluids, IV insulin, and potassium replacement. He did well clinically and his DKA improved. He was discharged on a basal/bolus regimen of insulin with metformin, with a plan to follow up in a specialized diabetes clinic.

**TABLE 1:** Laboratory parameters and characteristics of patient

Variable	Day 1 (admission)	Day 5 (discharge)	Follow‐up (day 58)
WBC (10^6^/μL)	14.2	10.8	6.8
Hemoglobin (g/dL)	16.7	13.6	13.7
Hematocrit (%)	53.3	40.6	40
Platelets (10^6^/μL)	299	291	390
CRP (mg/L)	245	76.9	10.1
Ferritin (μg/L)	1091	764	243
Creatinine	80	47	41
Alkaline phosphatase (units/L)	128	60	55
Interleukin‐6 (pg/mL)	36	–	26
Blood sugar (mmol/L)	6.3	8.2	7.9
B hydroxybutyrates (mmol/L)	6.56	0.06	–
Ethanol level	Negative	–	–
pH	7.21	7.42	–
pCO_2_ (mm Hg)	30	30	–
pO_2_ (mm Hg)	85	80	–
HCO_3_ (mmol/L)	14	20	24
Sodium (mmol/L)	143	136	135
Potassium (mmol/L)	4.8	3.8	4.6
Chloride (mmol/L)	111	108	102
Lactic acid (mmol/L)	1.1	1.3	1.8
Albumin (g/L)	22	20	26
Anion gap (mmol/L)	18	8	9
Delta gap (mmol/L)	6	−4	−3
Delta ratio	0.6	−1	–
Albumin corrected anion gap (mmol/L)	23.5	13	12.5
Albumin corrected delta gap (mmol/L)	10.5	1	0.5
Albumin corrected delta ratio	1.1	0.3	–

−, not available.

SGLT‐2 inhibitors have a pro‐ketogenic and calorie restriction effect due to the loss of glucose in urine. Chronic hyperglycemia can lead to insulin resistance. SGLT‐2 inhibitors play a role in decreasing insulin resistance by a decrease in serum glucose levels secondary to glucosuria. The decreased serum glucose load decreases the stimulus for beta cells to secrete insulin. Bonner et al demonstrated that alpha cells in the pancreas contain SGLT‐2 co‐transporters. Inhibition of these co‐transporters by dapagliflozin leads to an increased glucagon secretion by alpha cells. SGLT‐2 inhibitors may also decrease ketone bodies' excretion leading to ketosis with euglycemia.^3^ This decrease in serum glucose levels decreased insulin secretion, and decreased ketone bodies excretion may give rise to the clinical dilemma of euDKA, which constitutes ketoacidosis without hyperglycemia.

There is an ongoing phase III, multicenter, parallel‐group, randomized, double‐blind, placebo‐controlled trial (ClinicalTrials.gov Identifier: NCT04350593) on Dapagliflozin in respiratory failure in patients with COVID‐19 (DARE‐19). DARE‐19 aims to establish the role of dapagliflozin in reducing disease progression, complications, and all‐cause mortality. The authors are of the view that the use of SGLT‐2 inhibitors in critically ill patients with an underlying SARS‐CoV‐2 infection may raise several safety issues. Random blood glucose level is used as a surrogate marker for the body's insulin requirement. With the use of SGLT‐2 inhibitors, blood glucose may underrepresent the insulin requirement. This may lead to ketoacidosis development and contribute to significant morbidity and mortality.

Respiratory failure in DKA is associated with increased morbidity and mortality.^4^ Metabolic acidosis leads to a compensatory respiratory response. The initial tachypnea decreases CO2 concentration. Tachypnea is followed by hyperpnea, which increases tidal volume, followed by a deep, fast, and agonal breathing pattern called Kussmaul's respiration. Kussmaul breathing pattern is a harbinger for the development of respiratory fatigue and may lead to intubation and mechanical ventilation if not addressed in time. Furthermore, in a patient of COVID‐19 with euDKA, a normal serum glucose level coupled with anchoring bias may mislead the clinician in interpreting Kussmaul's breathing as COVID‐19 associated respiratory illness or one of its respiratory complications.

Our patient was labeled as severe COVID‐19 pneumonia‐causing respiratory compromise upon initial triage. The euDKA diagnosis became clear upon detailed history, including medication history and a blood gas analysis. Authors believe that during the COVID‐19 pandemic, there should be a cautious use of SGLT‐2 inhibitors. This caution is even more critical in patients with COVID‐19 pneumonia, as the use of SGLT‐2 inhibitors may lead to a misinterpretation of clinical signs and a resultant clinical error.

## CONFLICT OF INTEREST

The authors declare that there is no conflict of interest that could be perceived as prejudicing the impartiality of the research reported.

## AUTHOR CONTRIBUTIONS

Zohaib Yousaf contributed towards analyzing the case, writing the manuscript, and critically revised the manuscript to the final form. Waqar Munir contributed to the manuscript write up. All authors approved the final version for submission. Riyadh Hammamy was the treating consultant, obtained informed consent, and critically revised the draft.

## ETHICS APPROVAL

The project was approved by the ethics committee of Hamad Medical Corporation in accordance with the principles of the Helsinki Declarations and the guidelines of ministry of public health Qatar. The project ID assigned by the medical research council is MRC‐04‐20‐636. All patients signed the written informed consent.

## References

[1] Fralick M , Schneeweiss S , Patorno E . Risk of diabetic ketoacidosis after initiation of an SGLT2 inhibitor. N Engl J Med. 2017;376(23):2300‐2302. 28591538 10.1056/NEJMc1701990

[2] Ogawa W , Sakaguchi K . Euglycemic diabetic ketoacidosis induced by SGLT2 inhibitors: possible mechanism and contributing factors. J Diabetes Investig. 2015;7(2):135‐138. 10.1111/jdi.12401 PMC4773669 27042263

[3] Bonner C , Kerr‐Conte J , Gmyr V , et al. Inhibition of the glucose transporter SGLT2 with dapagliflozin in pancreatic alpha cells triggers glucagon secretion. Nat Med. 2015;21(5):512‐517. 25894829 10.1038/nm.3828

[4] Regmi A , Konstantinov N , Agaba E , Rohrscheib M , Dorin R , Tzamaloukas A . Respiratory failure in the course of treatment of diabetic ketoacidosis. Clin Diabetes. 2014;32(1):28‐31. 26246676 10.2337/diaclin.32.1.28 PMC4521429

